# Efficacy of Alkaloids in Alleviating Myocardial Ischemia-Reperfusion Injury in Rats: A Meta-Analysis of Animal Studies

**DOI:** 10.1155/2021/6661526

**Published:** 2021-03-19

**Authors:** Shuai Wang, Han Liu, Yang Zhang, Liqun Ren

**Affiliations:** ^1^Department of Experimental Pharmacology and Toxicology, School of Pharmaceutical Science, Jilin University, Changchun, Jilin, China; ^2^Department of Vascular Surgery, The First Hospital of Jilin University, Changchun, Jilin, China; ^3^Department of Respiration, The First Hospital of Jilin University, Changchun, Jilin, China

## Abstract

**Background:**

Animal models are well established for studying the effects of alkaloids in preventing myocardial ischemia-reperfusion injury. However, few studies have investigated the therapeutic effects of alkaloids in humans. This meta-analysis and systematic review assessed the efficacy of alkaloids in attenuating infarct size in rats with myocardial ischemia-reperfusion injury.

**Methods:**

An integrated literature search including the PubMed, Embase, and Cochrane Library databases was performed to identify studies that evaluated the therapeutic effects of alkaloids on myocardial ischemia-reperfusion injury in rats. The main outcome was infarct size, and SYRCLE's risk of bias tool was used to assess the quality of the studies.

**Results:**

22 studies were brought into the meta-analysis. Compared with the effects of vehicle, alkaloids significantly reduced infarct size (standardized mean difference (SMD) = −0.45; 95% confidence interval (CI) = −0.64 to − 0.26). In subgroup analyses, isoquinoline alkaloids (SMD = −0.43; 95%CI = −0.70 to − 0.16) significantly reduced infarct size versus the control.

**Conclusion:**

Isoquinoline alkaloids can potentially alleviate myocardial ischemia-reperfusion injury. This meta-analysis and systematic review supply a reference for research programs aiming to develop alkaloid-based clinical drugs. This trial is registered with CRD42019135489.

## 1. Introduction

Myocardial infarction (MI) constitutes the main cause of morbidity and mortality in patients with coronary heart disease globally [1, 2]. MI develops when there is a severe imbalance between myocardial oxygen supply and demand, resulting in myocardial hypoxia [1]. As the primary treatment, timely reperfusion plays a key role in the treatment of MI. However, a secondary cardiac injury called ischemia-reperfusion (I/R) injury can increase myocardial infarct size and induce a series of pathologic reactions, such as arrhythmia, left ventricular remodeling, and cardiac dysfunction [3–5]. Therefore, it is extremely necessary to develop novel prevention and therapy strategies.

In recent years, some studies illustrated that natural compounds, such as alkaloids, flavonoids, and terpenoids, attenuate myocardial I/R injury [6–8]. Alkaloids comprise a group of naturally occurring chemical compounds that mostly contain basic nitrogen atoms [9]. With a large range of pharmacological effects, such as antitumor, antiatherosclerotic, anti-inflammatory, and anti-I/R injury activities [10–12], they have been widely used in traditional and modern medicine [9]. However, most studies on the attenuating effects of alkaloids on myocardial I/R injury focused on isoquinoline alkaloids such as berberine and coptisine [13, 14], and a consensus on the effects of other alkaloids on myocardial I/R injury has not been reached.

Animal studies are conducted to investigate the safety and efficacy of novel interventions. Moreover, they are extremely valuable to build a bridge between basic research and clinical trials. In general, the rat is the most popular animal model for evaluating myocardial I/R injury induced through ligation of the left anterior descending artery [15]. To date, most systematic reviews and meta-analyses evaluated the effects of alkaloids on myocardial infarct size in rat I/R models in an effort to understand the clinical potential of alkaloids as anti-I/R injury agents. Thus, this systematic review and meta-analysis assessed the effects of alkaloids on infarct size in rats with myocardial I/R injury.

## 2. Materials and Methods

### 2.1. Reporting Standards

This systematic review conformed to the Preferred Reporting Items for Systematic Reviews and Meta-Analyses (PRISMA) statement, and the protocol was constructed using the SYRCLE format for animal intervention studies [16, 17].

### 2.2. Search Strategy

With the keywords “myocardial ischemia-reperfusion injury”, “rats”, and “alkaloid”, an experienced literature seeker (SW) searched the PubMed, Embase, and Cochrane Library databases for studies published between January 2001 and June 2019. In addition, the following search strategies were employed: (myocardial infarction OR myocardial ischemia OR myocardial I/R) AND (rat OR rats) AND (alkaloid OR aporphine OR belladonna OR indole OR isoquinoline OR opiate OR quinolizidine OR xanthine). A manual checking was performed to identify relevant studies from the reference lists of the included studies and review articles. The search was conducted on June 12, 2019, and limited among the articles published in English language.

### 2.3. Inclusion and Exclusion Criteria

The inclusion criteria were as follows: (1) original research, (2) rat as the animal model, (3) myocardial I/R injury as the disease model, and (4) alkaloids as the intervention. The exclusion criteria were as follows: (1) case reports, conference abstracts, review articles, editorials, and comments; (2) missing data; and (3) overlapping or duplicate datasets.

### 2.4. Study Selection

Two reviewers (HL and YZ) independently searched the titles and abstracts of the articles identified by the literature search to select eligible studies. The full text of potentially relevant articles was retrieved and independently examined by two reviewers (SW and LR) to determine whether these studies were in accordance with the inclusion criteria. Disagreements on study selection were resolved by discussion and consensus.

### 2.5. Data Extraction

Data extraction was performed by two reviewers (SW and HL) independently from the eligible studies. The first author's name, year of publication, age of rats, gender, diet, weight, alkaloid dose, time and route of treatment, duration of ischemia and reperfusion, and control and treatment group sample sizes were examined. Data that were showed graphically in the original publications were extracted using Adobe Photoshop 7.0.

The main outcome was infarct size measured as a numerical or percentage value. Disagreements on data extraction were resolved by discussion and consensus.

### 2.6. Quality Assessment

Quality assessment of the included studies was conducted by two investigators (YZ and LR) independently using SYRCLE's risk of bias tool, which contains domains evaluating sequence generation, baseline characteristics, allocation concealment, random housing, blinding, random and selective outcome assessments, incomplete outcome data, and other sources of bias [18]. Publication bias was detected via visual inspection of funnel plots. Disagreements on quality assessment were resolved by discussion and consensus.

### 2.7. Data Synthesis and Statistical Analysis

Statistical analyses were conducted using Review Manager (RevMan Version 5.3 for Windows Copenhagen: The Nordic Cochrane Centre, The Cochrane Collaboration, 2014). Standardized mean differences (SMDs) with 95% confidence intervals (CIs) were calculated to present the effects of vehicle and alkaloids on infarct size. A random-effects model was used to pool studies. Heterogeneity was categorized as moderate (*I*^2^ ≥ 30%) or high (*I*^2^ ≥ 50%) using the inconsistency index.

Multiple independent groups in a study (e.g., different alkaloid doses) were considered separate datasets. In nine studies [19–27], multiple groups investigating different alkaloid doses were compared to a single control group. To avoid an artificial increase in sample size in the pooled analysis, the number of animals in the control group for each study was divided by the number of comparator groups.

Subgroup analyses were performed to evaluate the effects of aporphine, belladonna, indole, isoquinoline, opiate, and other alkaloids on infarct size. Sensitivity analyses were performed to determine whether the findings were robust. *P* < 0.05 was considered statistically significant.

## 3. Results

### 3.1. Study Selection

**Figure 1:**
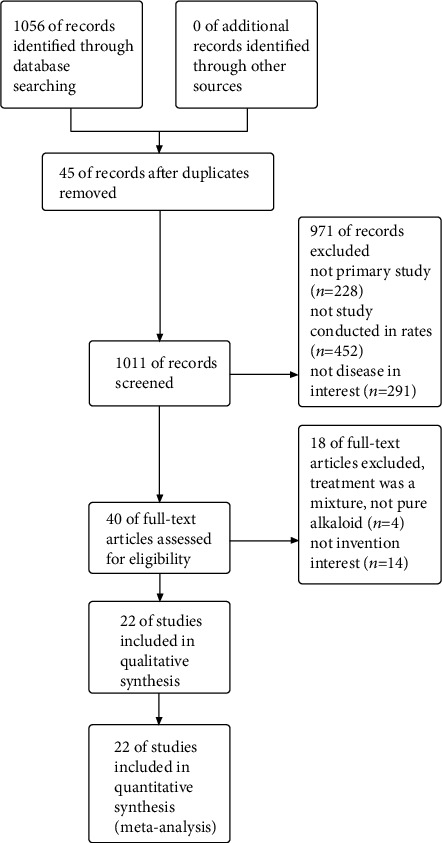
Flow diagram of the study identification and selection process.

In total, 1056 articles were identified in original search. After screening titles and abstracts, 40 studies were considered potentially eligible for inclusion. After estimating full-text publications, 14 studies were excluded due to intervention or outcome data were unqualified [28–41], and four studies were excluded because pure alkaloids were not assessed [42–45]. Finally, 22 studies were included in the present meta-analysis [13–14, 19–27, 46–56] (Figure 1).

### 3.2. Study Characteristics

The included studies contained 35 datasets, a total of 513 animals, and the characteristics are described in Table 1. The intervention was isoquinoline alkaloids in 10 studies [13–14, 19, 24, 47–48, 50, 54–56], opiate alkaloids in four studies [20–23], indole alkaloids in two studies [26, 53], aporphine alkaloids in two studies [46, 49], belladonna alkaloids in two studies [25, 27], and other alkaloids in two studies [51, 52].

Twenty-one studies used male animals [13, 14, 19–27, 46–50, 52–56], and the gender of the animals was not reported in one study [51]. Rats received a normal chow diet in 17 studies [13, 14, 19, 20, 22–27, 47, 49–52, 55, 56], and the diet was not reported in five studies [21, 46, 48, 53, 54]. Alkaloid administration was initiated in 8-week-old rats in five studies [19, 49, 51, 55, 56], 9-week-old rats in two studies [14, 48], 10-week-old rats in six studies [13, 21–23, 27, 52], and 12-week-old rats in one study [24], and animal age was not reported in eight studies [20, 25, 26, 46, 47, 50, 53, 54]. The weight of the rats varied from 200 to 350 g.

The route of alkaloid administration was intravenous in 11 studies [20–23, 25, 26, 46, 49, 51, 53, 56], intragastric in nine studies [13, 14, 19, 24, 27, 47, 52, 54, 55], and intraperitoneal in two studies [48, 50]. The duration of ischemia varied from 30 to 60 min, and that of reperfusion varied from 30 min to 24 h. Treatment was conducted prior to ischemia in 17 studies [13, 14, 19–22, 24, 25, 27, 47–52, 54, 55] and during ischemia in five studies [23, 26, 46, 53, 56].

### 3.3. Quality Assessment

**Figure 2:**
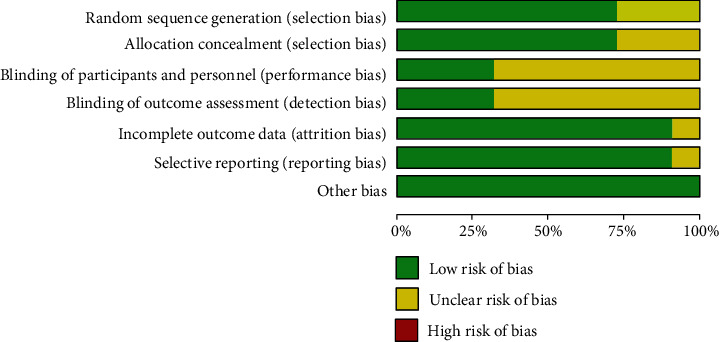
Risk of bias and quality assessment score (%) per risk of bias item.

**Figure 3:**
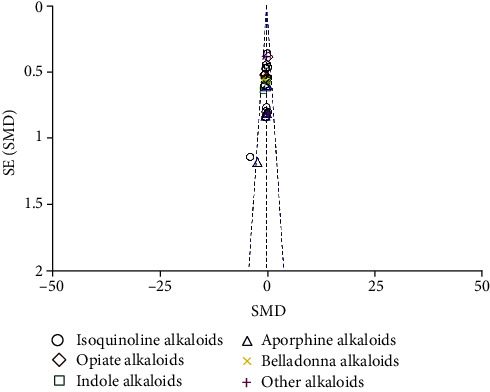
A funnel plot for evaluating publication bias.

The results of the study quality assessment are presented in Figure 2. In total, 16 studies were randomized. The risks of bias caused allocation concealment, and blinding was 72.7 and 31.8%, respectively. It showed no missing outcome data in twenty studies, and the risk of selective outcomes reporting was unclear in two studies. Across studies, the risk of bias from other sources was low. Visual inspection of the funnel plot indicated substantial publication bias (Figure 3).

### 3.4. Effect of Alkaloids on Infarct Size

**Figure 4:**
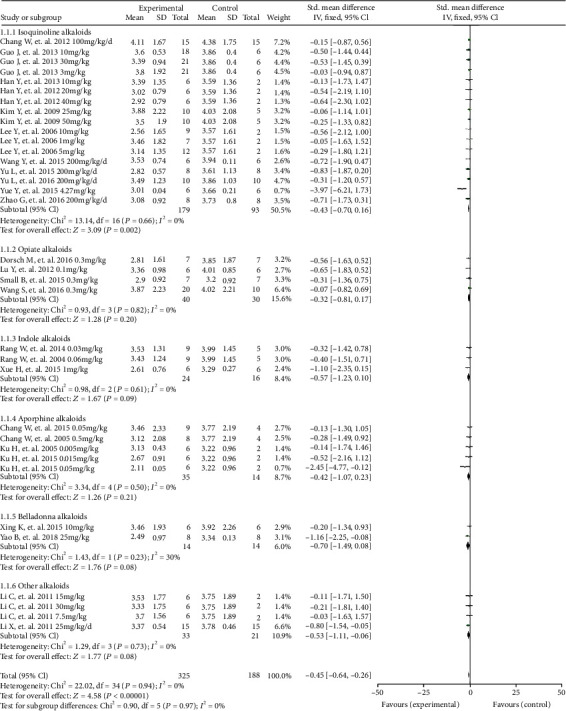
A forest plot of the effects of alkaloids on infarction size. Subgroup analyses evaluated the effects of isoquinoline, opiate, indole, aporphine, belladonna, and other alkaloids. SD: standard deviation; CI: confidence interval; Std: standard; IV: inverse variance.

The effect of alkaloids on infarct size was reported for 35 datasets obtained from 22 studies (alkaloid group, *n* = 325; vehicle group, *n* = 188). The meta-analysis indicated that compared with the effects of vehicle, alkaloids significantly reduced infarct size (*P* < 0.00001; SMD = −0.45; 95%CI = −0.64 to − 0.26). There was no evidence of heterogeneity between studies (*I*^2^ = 0%, Figure 4).

Subgroup analyses were performed to evaluate the effects of isoquinoline, opiate, indole, aporphine, belladonna, and other alkaloids on infarct size. Isoquinoline alkaloids (alkaloid group, *n* = 179; vehicle group, *n* = 93) significantly reduced infarct size versus vehicle (*P* = 0.002; SMD = −0.43; 95%CI = −0.70 to − 0.16), and there was no evidence of heterogeneity between studies (*I*^2^ = 0%). Opiate alkaloids (alkaloid group, *n* = 40; vehicle group, *n* = 30; *P* = 0.20; SMD = −0.32; 95%CI = −0.81 to 0.17), indole alkaloids (alkaloid group, *n* = 24; vehicle group, *n* = 16; *P* = 0.09; SMD = −0.57; 95%CI = −1.23 to 0.10), aporphine alkaloids (alkaloid group, *n* = 35; vehicle group, *n* = 14; *P* = 0.21; SMD = −0.42; 95%CI = −1.07, 0.23), and other alkaloids (alkaloid group, *n* = 33; vehicle group, *n* = 21; *P* = 0.08; SMD = −0.53; 95%CI = −1.11, 0.06) did not significantly decrease infarct size compared with the effects of vehicle, and there was no evidence of heterogeneity between studies (*I*^2^ = 0%). Belladonna alkaloids (alkaloid group, *n* = 14; vehicle group, *n* = 14) did not significantly reduce infarct size versus the control (*P* = 0.08; SMD = −0.70; 95%CI = −1.49 to 0.08), and there was a moderate degree of heterogeneity between studies (*I*^2^ = 30%).

**Figure 5:**
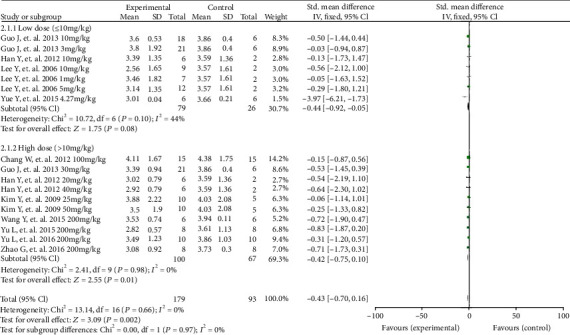
A forest plot of the effects of isoquinoline alkaloids on infarction size between high and low doses. SD: standard deviation; CI: confidence interval; Std: standard; IV: inverse variance.

In a subgroup analysis of isoquinoline alkaloids, high-dose (>10 mg/kg) treatment (alkaloid group, *n* = 100; vehicle group, *n* = 100) significantly reduced infarct size compared with the effects of vehicle (*P* = 0.01; SMD = −0.42; 95%CI = −0.75 to − 0.10), and there was no evidence of heterogeneity between studies (*I*^2^ = 0%). Low-dose (≤10 mg/kg) treatment (alkaloid group, *n* = 79; vehicle group, *n* = 26; *P* = 0.08; SMD = −0.44; 95%CI = −0.92 to 0.05) also significantly reduced infarct size versus vehicle, but there was evidence of moderate heterogeneity between studies (*I*^2^ = 44%, Figure 5).

The overall findings of the sensitivity analysis were not changed when a fixed-effects model was used instead of a random-effects model (SMD (95% CI): -0.45 (-0.64 to -0.26) vs. -0.55 (-0.67 to -0.43) and -0.43 (-0.70 to -0.16) vs. -0.55 (-0.69 to -0.42)).

## 4. Discussion

Based on previous animal studies, alkaloids have the potential to attenuate myocardial I/R injury. However, parameters such as the age and gender of animals, sample size, route type, and duration of ischemia and reperfusion varied among the studies. A quantitative synthesis and systematic analysis for animal study data that account for the sources of heterogeneity may have a potential to demonstrate the clinically desirable therapeutic efficacy of alkaloids in alleviating myocardial I/R injury. So we performed this systematic review and meta-analysis to evaluate the effects of alkaloids on myocardial I/R injury in rats. The findings illustrated that alkaloids significantly reduced myocardial infarct size versus vehicle.

Several alkaloid structures exist, including pyridines, isoquinolines, quinolines, indoles, purines, and aporphines [57]. In subgroup analyses, isoquinoline alkaloids significantly reduced infarct size compared with the vehicle in rats with myocardial I/R injury.

To our knowledge, this systematic review and meta-analysis is the first such study to estimate the efficacy of alkaloids in alleviating myocardial I/R injury in rats. The findings are potential to demonstrate a scientific basis for clinical studies of alkaloids in the treatment of myocardial I/R injury.

There was no heterogeneity between studies in the overall analysis, but there was a moderate degree of heterogeneity between studies in the analysis of belladonna alkaloids. Potential sources of heterogeneity include the duration of ischemia and reperfusion, each of which can progressively increase infarct size, and heterogeneity was found to be independently associated with both parameters [58].

This meta-analysis was associated with some limitations. First, it is necessary to discuss the relevance of our findings to humans due to species-specific differences in myocardial physiology. The rat myocardial I/R injury model is well established for studying myocardial ischemic disease. However, there are differences in pathogenesis. Specifically, the coronary arteries include the left anterior descending, circumflex, and right coronary arteries. All of these vessels can be attacked in patients with myocardial ischemic disease. However, only the left anterior descending artery was considered across studies. Second, only one parameter, infarct size, was used to estimate the efficacy of alkaloids in alleviating myocardial I/R injury. Other parameters, such as creatine kinase, cardiac troponin, heart rate, blood pressure, left ventricular developed pressure, and left ventricular end-diastolic pressure, were not considered [59–61]. Third, in this meta-analysis, other animal models of myocardial ischemia-reperfusion injury, such as mice, rabbits, and pigs, were not included. Fourth, the sample sizes of some included studies were relatively small. Further evaluation of animal studies with larger sample sizes is required to certify whether alkaloids are beneficial for alleviating myocardial I/R injury in humans.

The present meta-analysis and systematic review indicated that isoquinoline alkaloids have a clinical benefit on myocardial I/R injury in rats. Therefore, large scale, prospective, and well-designed animal studies are necessary to validate the mechanism of alkaloids in alleviating myocardial I/R injury. And more randomized controlled trials in humans are needed before the pharmacological therapeutic effects on myocardial I/R injury of alkaloids in patients are confirmed.

## Figures and Tables

**Table 1:** Characteristics of the included studies.

Study	Alkaloid	Age	Sex	Diet	Weight	Dose	Route	Treatment time	Ischemia duration	Reperfusion duration	Groups and sample size
Chang et al., 2005 [46]	Thaliporphine, aporphine	?	Male	?	250-350 g	0.05 mg/kg, 0.5 mg/kg	i.v.	During ischemia	60 min	2 h	Control = 8 Aporphine low = 9 Aporphine high = 8
Chang et al., 2012 [19]	Berberine, isoquinoline	8 W	Male	NCD	250-280 g	100 mg/kg	i.g.	Prior to ischemia	30 min	30 min	Control = 15 Isoquinoline = 15
Dorsch et al., 2016 [20]	Morphine, opiate	?	Male	NCD	300 g	0.3 mg/kg	i.v.	Prior to ischemia	30 min	2 h	Control = 7 Opiate = 7
Guo et al., 2013 [13]	Coptisine, isoquinoline	10 W	Male	NCD	260-300 g	3 mg/kg, 10 mg/kg, 30 mg/kg	i.g.	Prior to ischemia	30 min	3 h	Control = 18 Isoquinoline low = 21 Isoquinoline medium = 18 Isoquinoline high = 21
Han et al., 2012 [47]	Tetrahydropalmatine, isoquinoline	?	Male	NCD	250-300 g	10 mg/kg, 20 mg/kg, 40 mg/kg	i.g.	Prior to ischemia	30 min	6 h	Control = 6 Isoquinoline low = 6 Isoquinoline medium = 6 Isoquinoline high = 6
Kim et al., 2009 [48]	Palmatine, isoquinoline	9 W	Male	?	200-250 g	25 mg/kg, 50 mg/kg	i.p.	Prior to ischemia	30 min	6 h	Control = 10 Isoquinoline low = 10 Isoquinoline high = 10
Ku et al., 2015 [49]	TM-1-1DP, aporphine	8 W	Male	NCD	?	0.005 mg/kg,0.015 mg/kg, 0.05 mg/kg	i.v.	Prior to ischemia	60 min	2 h	Control = 6 Aporphine low = 6 Aporphine medium = 6 Aporphine high = 6
Lee et al., 2006 [50]	Higenamine, isoquinoline	?	Male	NCD	220-250 g	1 mg/kg, 5 mg/kg, 10 mg/kg	i.p.	Prior to ischemia	30 min	24 h	Control = 6 Isoquinoline low = 7 Isoquinoline medium = 12 Isoquinoline high = 9
Li et al., 2011 [51]	Sophocarpine, quinolizidine	8 W	?	NCD	230-270 g	7.5 mg/kg, 15 mg/kg, 30 mg/kg	i.v.	Prior to ischemia	30 min	2 h	Control = 6 Quinolizidine low = 6 Quinolizidine medium = 6 Quinolizidine high = 6
Li et al., 2011 [52]	Caffeine, xanthine	10 W	Male	NCD	200-250 g	25 mg/kg	i.g.	Prior to ischemia	30 min	3 h	Model control = 15 Xanthine = 15
Lu et al., 2012 [21]	Morphine, opiate	10 W	Male	?	280-300 g	0.1 mg/kg	i.v.	Prior to ischemia	30 min	2 h	Control = 6 Opiate = 6
Rang et al., 2004 [53]	Evodiamine, indole	?	Male	?	220-250 g	0.03 mg/kg, 0.06 mg/kg	i.v.	During ischemia	60 min	3 h	Control = 10 Indole low = 9 Indole high = 9
Small et al., 2015 [22]	Morphine, opiate	10 W	Male	NCD	?	0.3 mg/kg	i.v.	Prior to ischemia	30 min	2 h	Control = 7 Opiate = 7
Wang et al., 2016 [23]	Morphine, opiate	10 W	Male	NCD	280-320 g	0.3 mg/kg	i.v.	During ischemia	40 min	3 h	Control = 10 Opiate = 20
Wang et al., 2015 [24]	Berberine, isoquinoline	12 W	Male	NCD	250-300 g	200 mg/kg	i.g.	Prior to ischemia	30 min	2 h	Control = 6 Isoquinoline = 6
Xing et al., 2015 [25]	Anisodamine, belladonna	?	Male	NCD	250-300 g	10 mg/kg	i.v.	Prior to ischemia	45 min	1 h	Control = 6 Belladonna = 6
Xue et al., 2015 [26]	Evodiamine, indole	?	Male	NCD	220-240 g	1 mg/kg	i.v.	During reperfusion	30 min	2 h	Control = 6 Indole = 6
Yao et al., 2018 [27]	Anisodamine, belladonna	10 W	Male	NCD	200-250 g	25 mg/kg	i.g.	Prior to ischemia	60 min	2 h	Control = 8 Belladonna = 8
Yu et al., 2015 [54]	Berberine, isoquinoline	?	Male	?	220-250 g	200 mg/kg	i.g.	Prior to ischemia	30 min	6 h	Control = 8 Isoquinoline = 8
Yu et al., 2016 [55]	Berberine, isoquinoline	8 W	Male	NCD	250-300 g	200 mg/kg	i.g.	Prior to ischemia	25 min	2 h	Control = 10 Isoquinoline = 10
Yue et al., 2015 [56]	Coptisine, isoquinoline	8 W	Male	NCD	230-280 g	4.27 mg/kg	i.v.	During reperfusion	45 min	2 h	Control = 6 Isoquinoline = 6
Zhao et al., 2016 [14]	Berberine, isoquinoline	9 W	Male	NCD	200-250 g	200 mg/kg	i.g.	Prior to ischemia	30 min	6 h	Control = 8 Isoquinoline = 8

Note: NCD: normal-chow diet; i.g.: intragastric; i.p.: intraperitoneal injection; i.v.: intravenous injection; ? = not reported.

## Data Availability

The data used and/or analyzed during the current study are available from the corresponding author on reasonable request.
